# Microfluidic assessment of adhesion by surface display (MAPS-D): a novel method for evaluating peptide adhesion to polystyrene and poly(methyl methacrylate)

**DOI:** 10.1039/d5ra02983j

**Published:** 2025-09-17

**Authors:** Mark T. Kozlowski, Rebecca L. Renberg, Margaret M. Hurley, Jose A. Wippold, Justin P. Jahnke, Randall A. Hughes, Joshua A. Orlicki

**Affiliations:** a DEVCOM Army Research Laboratory, Army Research Directorate 2800 Powder Mill Road Adelphi MD 20783 USA joshua.a.orlicki.civ@army.mil

## Abstract

Many common polymers have low surface energies and limited chemical functionalities, making it difficult to promote adhesion to them without chemical or physical pre-treatment. This reduces the ability to conduct repairs of these materials at the point of need and limits their application. Biology already solves difficult adhesive problems and there may be novel biologically-derived adhesive mechanisms that can be used against these challenging substrates. However, biological materials such as peptides exist in a vast compositional space, can be expensive to synthesize, and techniques to evaluate their adhesive characteristics such as SFA, QCM, and SPR are often low-throughput. The Microfluidic Assessment of Adhesion by Surface Display (MAPS-D) technique is a semi-quantitative, on-cell, fluidics-based method to compare the ability of peptides to promote adhesion to substrates, demonstrated using polystyrene (PS) and poly(methyl methacrylate) (PMMA). Performance of the on-cell peptide was also compared to free-peptide and found to correlate well provided that more than 60% of evaluated cells successfully displayed the peptide of interest. It was also found that the number of cells that remained on the surface was dependent on flow rate, suggesting a “releasing force” could be calculated. This MAPS-D technique does not require expensive equipment, removes the need to synthesize and purify peptides, and has the potential to be made higher-throughput.

## Abstract 2

This work presents a novel method for evaluating the adhesive properties of peptides with higher throughput using a microfluidics approach.

## Graphical abstract

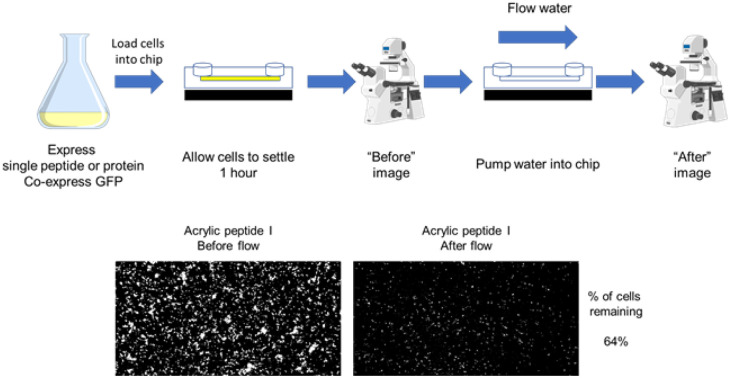

## Introduction

Polymers are ubiquitous in the modern world, with global production estimated at approximately 400 million metric tons in 2022.^1^ Most of this production consists of commodity plastics such as polyethylene terephthalate, polypropylene, polystyrene, and acrylic.^2^ Some of these polymers are characterized by low surface energy and high hydrophobicity, making them difficult to functionalize or bond to without heat or mechanical/chemical pre-treatment.^3^ This adds complexity and expense to manufacturing processes and limits the ability to repair polymeric components at point of need. Adhesive challenges remain for some additive manufacturing approaches, as poor interlayer bonding can lead to weaker mechanical properties of the finished product.^4–6^ Accumulation of defects at an interface (*e.g.* moisture infiltration, debonding) often limits the lifetime of an adhesive. Identifying new compositions to improve interfacial interactions could potentially improve the lifetime of adhesives in adverse conditions.

Biological systems have evolved to solve an array of adhesion challenges under the wide range of conditions present on Earth, including reversible adhesion in wet conditions^7,8^ which remains a challenge for synthetic adhesives. For this reason, study of biological and biomimetic adhesion has been quite extensive. Peptides for promotion of adhesion to polymer substrates have been found through various types of screening,^9–13^ as well as designed *de novo*.^14–16^ By studying peptide adhesion it may be possible to find completely novel binding modalities that have not yet been discovered. However, there are an enormous number of potential peptides to evaluate and even *de novo* design requires numerous peptides to be tested in order to validate and improve the models. A high-throughput screening method could facilitate the construction of reliable AI–ML models, which may prove invaluable in unlocking new insights about designing interactions for bulk substrates.

Current methods for measuring peptide adhesion to a surface include surface force analysis (SFA), quartz crystal microbalance (QCM) or surface plasmon resonance (SPR), powerful techniques which can give precise information about the binding affinity of a peptide.^17–19^ However, these processes require considerable training and expertise, and can require hours to run a single peptide. Furthermore, peptides need to be synthesized and purified before they can be run on QCM, SPR, or SFA, which adds additional cost and complication, and is not feasible if hundreds or thousands of peptides are to be evaluated. Macroscopic methods of adhesion testing, including peel or lap shear testing, are not suitable for evaluation of novel peptides as they require massive amounts of material relative to the tens or hundreds of milligrams of protein that can be produced without optimization. A very promising assay was recently developed by Griffith and co-workers whereby peptide gels were placed in a multiwell plate along with fluorescent beads: the plate was then inverted in a centrifuge, and the degree to which the beads were removed indicated whether a given peptide was strongly or weakly adhesive.^20^ This is a promising high-throughput assay but still has the disadvantage of requiring synthesis and purification of large numbers of peptides, which is expensive.

Development of an assay with sufficient throughput, fidelity, and lenient physical requirements could enable selection of peptides of greatest interest from a large library, and this smaller selection could then be subjected to more-precise measurements such as QCM, SPR, or SFA. This would fill an important gap on the continuum between high-throughput and low-fidelity assays (biopanning) and low-throughput but high-fidelity assays (QCM, SPR, SFA). Being able to rationally select from a large library would also enable the technique of directed evolution to be applied to the problem of biologically-inspired adhesion. Directed evolution is a method of protein discovery that involves making random mutations to a protein or peptide that has displayed some affinity for a particular process, such as enzymatic catalysis. The most promising mutations are selected for further mutation and analysis while the poorer mutants are discarded. Through this experimenter-directed selection it is possible to improve the function of extant proteins^21^ and to make enzymes that mediate reactions that are uncommon in nature, such as the Diels–Alder reaction.^22^ The potential of directed evolution to solve complex problems was recognized when development of the technique won the Nobel Prize in 2018.^23,24^ However, directed evolution requires the ability to analyze many proteins and mutant variants quickly. A higher-throughput, semi-quantitative, and cost-effective method for evaluating peptide and protein adhesion is therefore needed before directed evolution studies can even be contemplated.

To provide such an evaluation method, the current work combines two well-known techniques: cell surface display, and measurement of cellular adhesion in a microfluidic device. Cell-surface display is a well-established method for evaluating protein function, conducting biocatalysis and biosensing, and altering cellular behavior.^25–27^ It has the advantage of not requiring purification of the proteins, saving time and cost. Surface display also allows rapid mutations to be made to the proteins or peptides of interest, enabling a large number of variants to be scanned with the proper assay. Microfluidics technology concerns itself with the processing, manipulation, and analysis of small amounts of fluids, and is increasingly used in life sciences research because of its ability to derive a large amount of information from small samples with a high degree of sensitivity and specificity. Confinement of cells in microfluidic devices can also result in unique biophysical phenomena. Microfluidics can also be multiplexed and automated, enabling many replicates to be run in a short period of time.^28–31^ Pre-fabricated microfluidics can also be used in resource-limited settings by minimally-trained personnel, enabling a democratization of both research and medical and environmental testing.^32,33^ The basic architecture of a microfluidic chip can be placed on an arbitrary substrate of interest, and the modularity of microfluidic devices enables rapid prototyping, customization, and adaptation to diverse applications.^34,35^ Microfluidic approaches for studying the forces between cells and various surfaces are already well-established.^36–40^ A microfluidics-based approach is therefore attractive as a potential method for quick and simple evaluation of adhesion, as well as down-selection of peptides of interest. There are several commercial microfluidic chips available on the market, such as those produced by Ibidi that are used in this work, meaning that microfluidic techniques are increasingly available to non-specialized labs.

In sum, cell surface display allows a large number of variants to be screened, and in combination with microfluidics techniques provides for rapid screening. In this work, a microfluidics-based method was developed for screening the adhesive interactions of peptides displayed on the surface of bacterial cells, and demonstrated against model substrates polymethylmethacrylate (PMMA) and polystyrene (PS). These materials were chosen as models because of their widespread usage, difference in surface hydrophobicity, and the robust body of literature studying each. The peptides were also tested at four different flow rates, which correlates to four different shear rates, and it was found that the Stokes drag force required to remove the cells decorated with peptides could be estimated. This method obviates the need to synthesize and purify individual peptides and requires no scale-up of peptide expression. The semi-quantitative comparisons afforded by these methods aids in the down-selection of peptides from a larger library, and would also allow mutagenesis studies to be conducted, allowing some elucidation of the mechanism of adhesion.

## Methods and materials

### Cloning

The cloning strategy is detailed in a drawing in the SI (Fig. S1). In brief, a library of approximately 250 000 random 15-mer peptides was generated and expressed on the surface of *Escherichia coli* using the autodisplay/autotransporter surface display system.^41^ The expressed peptide had a His-tag (six histidine residues, HHHHHH) on its N-terminus, followed by a 15-amino acid variable region providing the compositional space that was evaluated. C-terminal to this 15-amino acid variable region was a twenty amino acid spacer (GS)_10_, and C-terminal to the (GS)_10_ spacer was the autotransporter protein embedded in the cell's outer membrane. The His-tag provided an epitope which allowed for confirmation of surface display of the peptide, and the (GS)_10_ spacer ensured that the variable region was not immediately adjacent to the cell. The overall length of the peptide including cloning scars is 41 residues. Each individual cell displayed a single peptide. DNA encoding this random peptide library was obtained from Integrated DNA Technologies (IDT, Coralville, IA), with appropriate restriction sites to enable cloning into an autotransporter surface-display construct. To remove stop codons present in the as-received library that would result in a sequence that would code for an incomplete, non-functional protein (a nonsense sequence), the library was first cloned into the custom-designed plasmid pFES2.AB in the middle of a split-intein ampicillin resistance cassette. If the DNA for the peptide did not contain a stop codon the ampicillin resistance cassette was complete and cells were able to propagate on agar medium containing ampicillin. When a stop codon was present in the DNA sequence, the cassette was incomplete, ampicillin resistance was not conferred and the cells died. Peptide sequences containing stop codons were therefore removed from the library. This reduced library without stop codons was then cloned into a second plasmid for surface display, as previously described.^42^ The peptides found by our study were compared to PS binders found by the teams of Kogot,^9^ Qiang,^11^ Vodnik,^12^ Woo,^14^ and Feng,^13^ and PMMA binders found by the teams of Iwasaki,^15^ and Waku.^16^ Surface-display constructs with these literature peptides were also cloned for comparison with library peptides found by biopanning.

### Biopanning

**Fig. 1:**
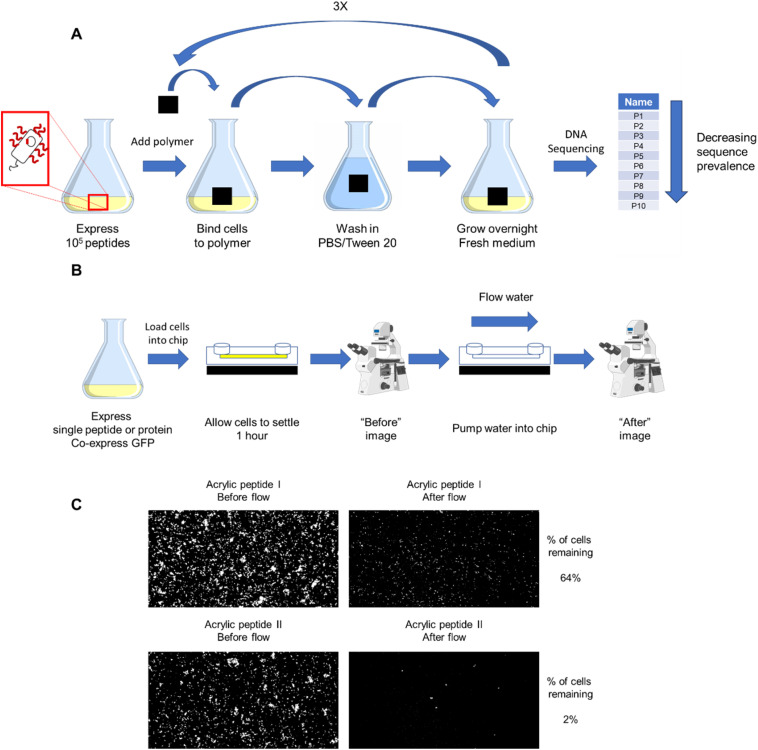
Experimental schematic for biopanning (A) and MAPS-D (B) experiments. Biopanning for promising peptides is illustrated in (A): cells were first cultured and induced to display peptides on their surface, with each cell expressing a unique peptide. A small coupon of material (polystyrene or PMMA) was then incubated with cells. The coupons were then washed to remove any cells that adhered weakly, then placed in fresh medium to propagate remaining cells. After 4 rounds of screening, the resulting library was subjected to next-generation sequencing, giving the prevalence of what peptides remain. The top 10 most-prevalent peptides found in the sequencing were isolated for further study. (B) Illustrates the MAPS-D technique. Cells with the DNA for a single peptide were grown and peptide expression was induced, green fluorescent protein (GFP) was constitutively expressed on a second plasmid. The cells were allowed to settle in the Ibidi chip, and then water was pumped into the chip to remove the cells. A fluorescent microscope was used to image the cells in the channel before and after flow. (C) Shows representative images of before and after images obtained in this technique.

To find an initial set of peptides for further analysis the biopanning approach of Stellwagen and co-workers was adapted,^10^ illustrated in Fig. 1A. To conduct the first round of substrate screening cells were grown overnight at 37° in lysogeny broth (LB, MilliporeSigma, Milwaukee, WI). To screen the libraries against polymers of interest, 1 mL of frozen stock was grown in a glass shaker flask at 37 °C at 250 rpm to an OD_600_ of 0.5 in 50 mL of LB medium supplemented with 30 mg L^−1^ of chloramphenicol. Library expression was induced with a final concentration of 0.1% l-arabinose and allowed to proceed overnight. The following morning two coupons of material approximately 1 cm by 1 cm by 0.3 cm (length–width–height) in size and made of acrylic or polystyrene were introduced to the culture (polymers supplied by McMaster-Carr, Elmhurst, IL). The coupons were incubated for 15 min at 37 °C at 225 rpm. The medium was removed and replaced with 50 mL of PBS supplemented with 1% (v/v) Triton X-100 (Sigma Aldrich). The coupons were washed for 30 min at 37 °C at 225 rpm in the PBS-Triton X-100. The coupons were removed and placed in 50 mL of LB medium supplemented with 30 mg L^−1^ of chloramphenicol and 2% d-glucose (to repress the araBAD promoter). This new inoculum was grown overnight at 37 °C. The following day the resulting culture was centrifuged for 6 min at 5000*g* and resuspended in 2.5 mL of LB medium supplemented with 25% glycerol. The resulting pellets were frozen in liquid nitrogen and kept at −80 °C for future use.

### Sequencing protocol and analysis

Plasmids were isolated from a frozen stock (round 4 of biopanning) for each sample using the ZymoPURE Plasmid Miniprep Kit (Zymo; Cat. No. D4210). Short-read sequencing libraries were prepared according to the Illumina 16S amplicon protocol using custom forward (ATlibSeq_For = TCGTCGGCAGCGTCAGATGTGTATAAGAGACAGATTGTTATTACTCGCGGCCCAGC) and reverse (ATlibSeq_Rev = GTCTCGTGGGCTCGGAGATGTGTATAAGAGACAGACCGGAACCAGAGCCAGAAC) primers (Illumina platform adapter overhangs and multiplex barcodes not shown) corresponding to autotransporter scaffold regions adjacent to the peptide insert. Final sequencing libraries were sequenced PE 2 × 151 bp on an iSeq 100 (Illumina; Santa Cruz, CA) with a 10% PhiX spike-in. Each sample generated 1.7–2.1 million reads. Illumina fastq files were analyzed using modified Matlab scripts based on the format presented by Matochko and co-workers in 2012.^43^

Experimental fastq files were searched for the correct flanking information and insert DNA sequences were extracted into a separate file. Biopython was used to translate this list of sequences from DNA to amino acid. The list of inserts was filtered to remove sequences containing frame shifts, unknown amino acids, stop codons, and any insert not containing exactly 15 amino acids. Processing and analysis was performed with a combination of bash, awk, biopython, and numpy. The ten most frequently occuring peptide sequences after four rounds of screening were identified for each substrate, cells with those sequences were isolated, and evaluated individually for their ability to promote adhesion to PS and PMMA as shown in Fig. 3B and 4B.

### Constant flow rate affinity assay

For the flow-based assessment of peptide adhesion as illustrated in Fig. 1B, surface-display plasmid constructs for the individual peptides of interest were double-transformed with a second plasmid that constitutively expressed green fluorescent protein (GFP), enabling the cells to be tracked with fluorescent imaging (the sfGFP plasmid was a generous gift of Dr Nathan Schwalm of ARL). The cells were cultured and expression of the surface-display peptide was induced as described for the full library. A commercially-available microfluidic chip (sticky-Slide I Luer Slide, 0.1 mm gap, Ibidi, Fitchburg, WI) was adhered to a PMMA or PS substrate using the adhesive provided on the chip. An aliquot of cells in media (OD_600_ 0.3, ∼500 μL) was injected into the channel of the chip, and the cells were allowed to settle for one hour without flow. A microscopic image was taken using a Panthera C2 microscope with green fluorescence module (Motic, San Antonio, TX) of a point in the flow channel in the center of the chip (10× magnification, field of view approx. 2 mm × 2 mm). Water was then flowed into the chip at a constant rate of 70 μL min^−1^ for five minutes; according to the manufacturer's guidelines these conditions provided a shear stress of 0.6 dynes per cm^2^ or 0.06 Pa. A second image was then taken in the same spot: the chip contained a circular index marking in the center, and this was used to ensure that the same spot was imaged before and after flow. The number of cells present in the before and after images was determined using ImageJ software (National Institutes of Health, Bethesda, MD).^44^ Comparison of the cell counts before and after flow yielded the fraction of cells remaining, presented for each substrate in Fig. 3A and 4A. The flow rate of 70 μL min^−1^ was chosen for all experiments because initial surveys showed that at this flow rate, almost all of the fluorescent *E. coli* cells that did not display a peptide were removed within a minute of starting flow (data not shown). Additional details are found in the SI.

### Variable flow rates

Monticelli and co-workers developed a magnetic-based method to exert force on cells in a microfluidic device, which could be used to calculate the amount of adhesive force that a cell was capable of exerting.^45^ It was hypothesized that a similar method for estimating the binding strength of the peptides could be developed by using varying flow rates in a microfluidic device. The Stokes drag was calculated for four different flow rates. First, the Reynolds number for the variable flow rates was calculated using eqn (1) to determine the appropriate drag force equation for the experimental volumetric flow rates of 30 μL min^−1^, 70 μL min^−1^, 140 μL min^−1^, and 210 μL min^−1^.1
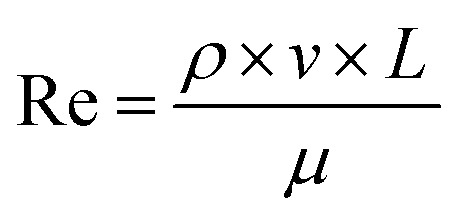
where Re is the resultant Reynolds number, *ρ* is the fluid density, *v* is the flow velocity, *L* is the characteristic length, and *μ* is the dynamic viscosity. For this calculation, the fluid dynamic viscosity (*μ*) was assumed to be that of water (0.001002 Pa s) as the flow-through solution was water which rapidly replaced the medium in the flow channel. For each calculation, the volumetric flow rate from the driving force (*e.g.* syringe pump) was converted to flow velocity using eqn (2);

2 *Q* = *v* × *A*

where *Q* is the volumetric flow rate, *v* is the flow velocity, and *A* is the channel area. The characteristic length used for these calculations, given that the microfluidic system was comprised of rectangular profile microchannels, was the hydraulic diameter of the flow channel as seen in eqn (3)3
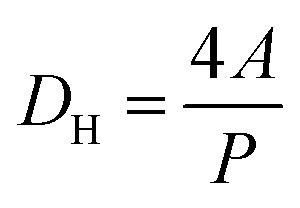
where *D*_H_ is the hydraulic diameter, *A* is the microchannel's cross-sectional area, and *P* is the perimeter. For all calculations, the channel area was derived using the dimensions supplied by the microfluidic chip manufacturer. The microfluidic chip height was 0.15 mm (150 μm) and width of 10.25 mm (10 250 μm). For all calculations, the cell was assumed to be a rod with a face diameter of 1 μm and length of 23 μm. Given values Re values < 1, a low-Reynolds-number drag formulation suitable for a slender body in confined Stokes flow was employed as seen in eqn (4).4
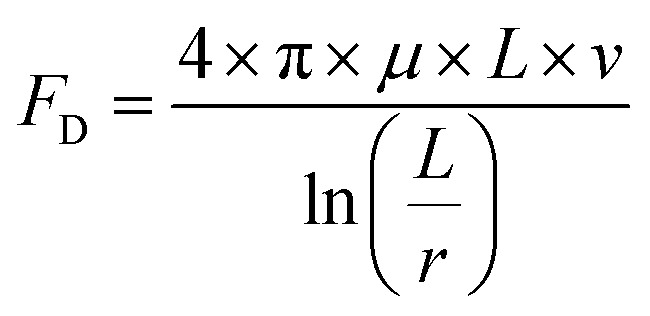
where *F*_D_ is the drag force, which in this low Reynold's number environment is characterized as the Stokes drag, *μ* is the dynamic viscosity, *L* is the length of cell, *v* is the flow velocity, and *r* is the radius of the cell. It must be noted that this equation has been adapted from the classical Stokes solution for flow around a cylinder to account for the confined microfluidic environment.

The Reynolds number calculated ranged from 0.096 (30 μL min^−1^) up to 0.67 (210 μL min^−1^). Given that even the upper value was below 1, the Stokes law was therefore used to calculate resultant drag force on the cells. The cells are anisotropic, and orientation of the cells cannot be controlled, meaning that some cells had their long ends aligned with the flow, while others had their long ends perpendicular to the flow. Therefore, a range of Stokes drag forces are presented: the low end of the range represents cells aligned parallel to the flow, the high end of the range represents cells that are perpendicular to the flow, and the actual force experienced by a particular cell lies in between these values. For the 30 μL min^−1^ condition, Stokes drag force ranges from 2.953 pN to 5.908 pN. For the 70 μL min^−1^ condition, Stokes drag force ranges from 6.892 pN to 13.78 pN. For the 140 μL min^−1^ condition, Stokes drag force ranges from 13.78 pN to 27.57 pN. For the 210 μL min^−1^ condition, Stokes drag force ranges from 20.67 pN to 41.35 pN.

### Flow cytometry

**Fig. 2:**
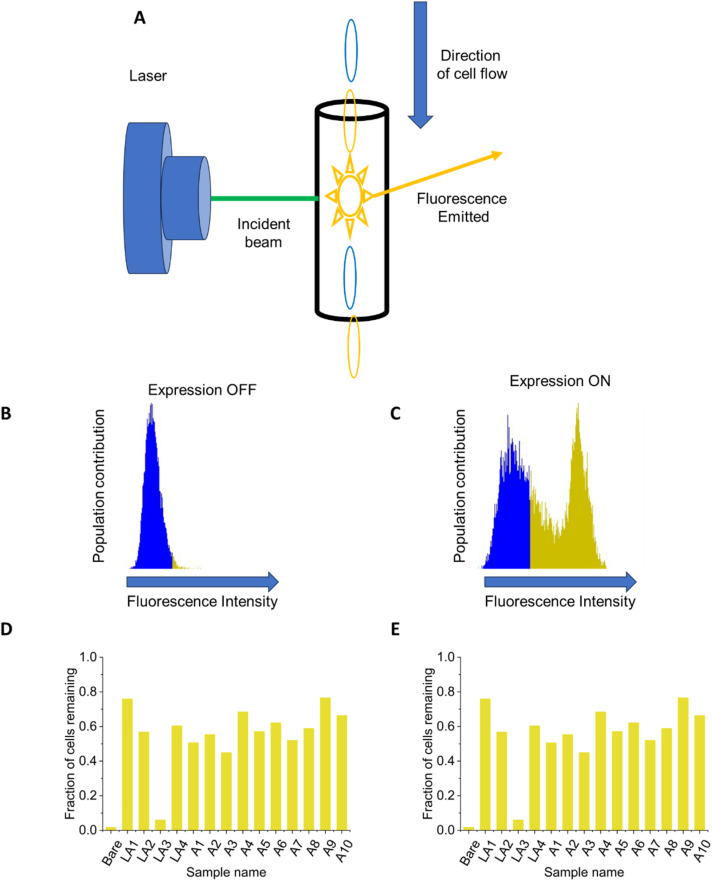
Flow cytometry for normalization. Flow cytometry was conducted to determine the number of cells which displayed a peptide, which is used to normalize the data from the Ibidi chips. 10 000 cells were analyzed for each sample. (A) Is a cartoon illustration of flow cytometry: a series of cells, some of which are fluorescent (red) and some of which are not (black) pass through a microfluidic channel where they are interrogated individually by a laser. The emitted fluorescence can be read to create charts such as those in (B and C). In (B and C), the *X* axis represents fluorescent signal in arbitrary units, and the *Y* axis represents the number of cells at each level of fluorescence. (B) Shows the cytometry for a set of cells for which peptide expression was not induced. This is used to set a threshold value for fluorescence as shown with an induced sample in (C): cells below the threshold are shown with blue shading, whereas cells that exceed that threshold are in gold shading. The percentage of cells that exceed the set threshold for each peptide is shown in (D) for putative polystyrene binders, and in (E) for putative PMMA binders, with the *X* axis showing the code for each peptide and the *Y* axis showing the percentage of cells exceeding the threshold. The bars shown in (D and E) represent single experiments. The sequences for each peptide are given in Fig. 3 and 4.

To account for possible differences in efficiency of peptide display, flow cytometry^46^ was used to establish which peptides displayed on the surface of the cells by analyzing a representative population of each library member. The surface display constructs were designed with a 6× histidine tag which could be recognized by a commercially-available antibody functionalized with Alexa Fluor 488 fluorescent dye. Only the cells expressing a surface-displayed peptide were stained by this antibody, and therefore the percentage of cells that were stained should be the same as the percentage of cells that display the peptide. The control consists of cells that have the antibody introduced but have not had the expression of the peptide induced. To analyze the results a threshold was imposed as shown in Fig. 2A such that the control experiment cells were below the threshold. The cell density and solution volume was kept constant, so the fluorescence threshold was valid across all evaluated peptides, and the percentage of cells that exceeded the threshold is reported in Fig. 2C and D for PS and PMMA respectively.

Individual constructs were transformed into *E. coli* strain BL21 by electroporation and spread onto LB-agar plates supplemented with 30 mg L^−1^ chloramphenicol. Individual colonies were chosen using a sterile pipette tip and were grown overnight at 37 °C in 5 mL LB medium supplemented with 30 mg L^−1^ chloramphenicol. The following morning, 5 mL of fresh cultures of LB medium supplemented with chloramphenicol were inoculated with 200 μL of overnight culture. The samples were allowed to incubate at 37 °C and 250 rpm until they reached an optical density (OD_600_) of approximately 0.5. A 2 mL aliquot of culture was removed to serve as an uninduced negative control, and the remainder was induced to a final concentration of 0.1% sterile-filtered l-arabinose. Both cultures were allowed to grow for an additional hour. Then, 1 mL of each culture was centrifuged at 4000*g* for 8 minutes, and then washed once with 1 mL of ice-cold phosphate-buffered saline (PBS; Sigma Aldrich) supplemented with 1% by weight of bovine serum albumin (BSA; Fisher Scientific). This solution was kept at 4 °C overnight. The following day, 25 μL of cells were antibody-stained by mixing with 25 μL of PBS-1% BSA containing 40 μg mL^−1^ of anti-6× His antibody conjugated to Alexa Fluor 488 dye, making 20 μg mL^−1^ of the final antibody concentration used for staining. The antibody was obtained from Thermo Fisher Scientific. The cells were allowed to stain for 1 hour at 4 °C. The cells were then washed twice with cold PBS (1 mL). The samples were then diluted 50-fold and evaluated on a Sony SA3800 spectral cell analyzer (Sony Biotechnology, San Jose, California). The uninduced negative control was used to threshold the induced samples.

### Free peptides

**Fig. 3:**
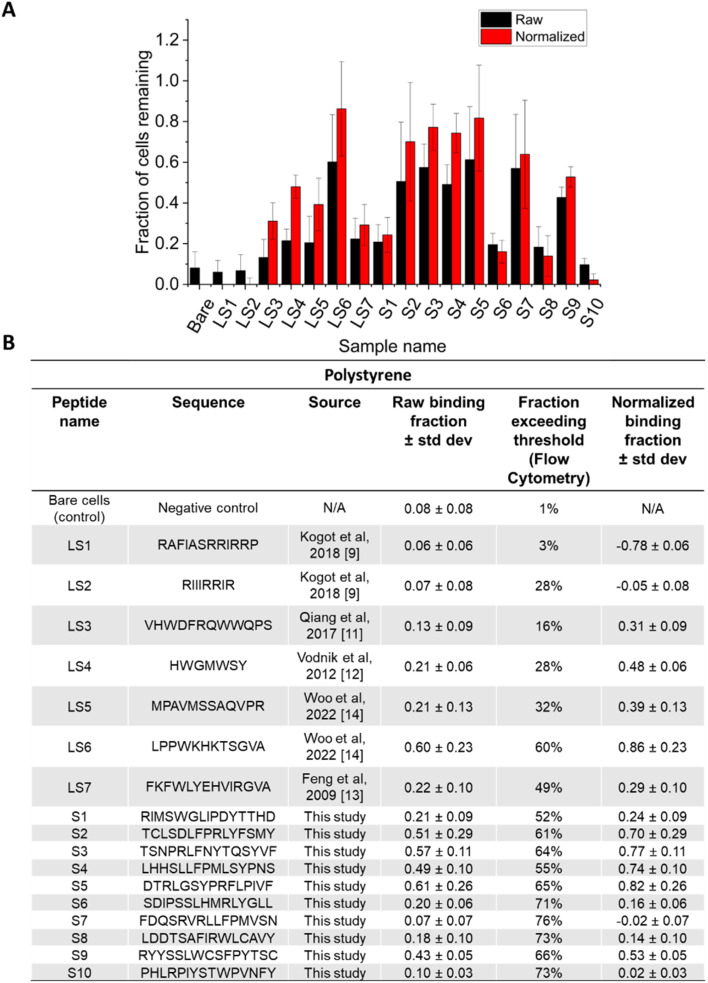
Polystyrene binding evaluated by MAPS-D. The ten most-common peptides from the initial library screen against polystyrene are titled S1–S10. These peptides, along with peptides obtained from literature (LS1–LS7) were evaluated using Ibidi microfluidic chips. (A) Shows raw, non-normalized data in black bars, and normalized data in red bars. (B) Shows the amino acid sequences of all peptides, as well as their source. The average of five technical replicates are shown, and error bars represent standard deviation. The flow rate was 70 μL min^−1^.

The peptides LS2, LS6, S3, S5, S7 and S10 (sequences provided in Fig. 3B) were ordered from Peptide 2.0 (Chantilly, VA). Each peptide was modified with a C-terminal FAM fluorescent dye enabling it to be tracked. The peptides were 15 amino acids in length, corresponding to the variable region of the peptides on-cell and not containing the GS spacer or His tag found in the bacterial cell constructs. The peptides were dissolved at a concentration of 0.5 mg mL^−1^ in ddH_2_O. The same polystyrene material as used previously in the MAPS-D experiments was procured and cut into rectangular coupons of approximately 12 cm by 18 cm. Prior to performing the spot assay, the surface of the coupon was cleaned by spraying with 70% ethanol and rubbed with paper towel and allowed to dry. Three spots of 50 μL each of peptide solution were placed on the surface in columns and rows. The spots were allowed to remain on the coupons at room temperature for 10 minutes before being removed by pipetting and gently dabbed dry with a Kimwipe. A “before wash” image was taken using an iBright 1500 imager (Thermo Fisher) imaging in the GFP channel, with an exposure time of 50 ms. The coupons were then put in a Pyrex baking tray which contained approximately 10 cm of DI water, and immersed. The baking tray was placed on a rotary table (Model SI-1700, Scientific Industries Inc., Bohemia, NY), which was then turned on at a speed of 40 rpm, with orbital shaking. The coupon was washed in this manner for 5 minutes, removed, dabbed dry, and imaged as before. The washing was continued for two additional 5-minute intervals, giving timepoints after 5-, 10-, and 15-minutes total. All images were exported as raw TIFF files and analyzed using ImageJ. In ImageJ, identical regions of interest were defined for each observed spot, and the “measurement” function was used to derive the integrated density (IntDen) of the fluorescent signal at each point. The IntDen of each spot after washing was divided by the IntDen of each spot before washing, to give a percentage of signal remaining. These results are shown in Fig. 6.

## Results and discussion

### Evaluation at a constant flow rate

As shown in Fig. 1A, the initial biopanning screens were conducted with small (1 cm × 1 cm × 0.3 cm) coupons of PMMA and PS that were introduced into a culture of *E. coli* cells that expressed a total of ∼250 000 different peptides on their surface. If a peptide showed some affinity for the substrate of interest, it would adhere the cell to that substrate, and the cell would remain on that substrate after washing. Cells remaining on the coupons after washing were then propagated by transferring the coupons to fresh medium. Four cycles using this method enriched the library for those surface-display peptides that had some degree of affinity for the substrate. Next-generation sequencing of these enriched libraries enabled the extraction of substrate-specific 15 amino acid peptide sequences, along with their relative abundance in culture. The ten most abundant peptides present in the expression library after the final round of biopanning were selected for further study and were labelled A1–A10 (PMMA binding enriched) and S1–S10 (PS binding enriched). Peptides that had previously been reported in the literature as promising binders for PMMA and PS were also studied, and are labelled LA1–LA4 and LS1–LS7 respectively. The 20 peptides discovered by biopanning, and the 11 peptides previously reported in the literature, were then evaluated with a novel method: Microfluidic Assessment of Adhesion by Surface Display (MAPS-D), which is illustrated in Fig. 1B. The ability of the cells to remain adhered to the substrate while being subjected to a shear force in a microfluidic chip at a constant flow rate of 70 μL min^−1^ was used as a proxy for the strength of the adhesive interaction between the peptide and the substrate. Water was used as the “purge” fluid in the current work, but this approach is also amenable to use of different solvents or buffers. The MAPS-D approach was validated by testing several peptides that had previously been reported to adhere to PMMA and PS in addition to the novel peptides found through biopanning screens.

Flow cytometry was used to determine the degree to which each peptide of interest successfully displayed on a bacterial surface, and to normalize MAPS-D results relative to the number of cells in each population that successfully displayed a peptide. Flow cytometry was used to obtain a histogram of cell fluorescence, which correlated to the amount of peptide displayed on the surface. Example cytometry histograms are shown in Fig. 2B and C, where the fluorescence intensity is shown on the *X* axis and the number of cells displaying a given fluorescence intensity is shown on the *Y* axis. A threshold based on the uninduced cell was then drawn, above which the cell was assumed to have peptide present. Based on this standard, the flow cytometry indicated that certain peptides such as LS1 displayed minimally, and that the best-displaying peptides expressed as intended on between 60% and 80% of cells. Five of the literature peptides (LS1, LS2, LS3, LS4, LS5, LA3) have fewer than 30% of the cells expressing the peptide on the surface. MAPS-D data were normalized using eqn (5):5
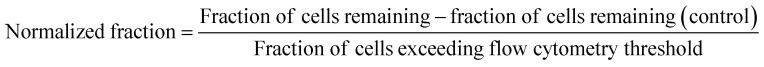


**Fig. 4:**
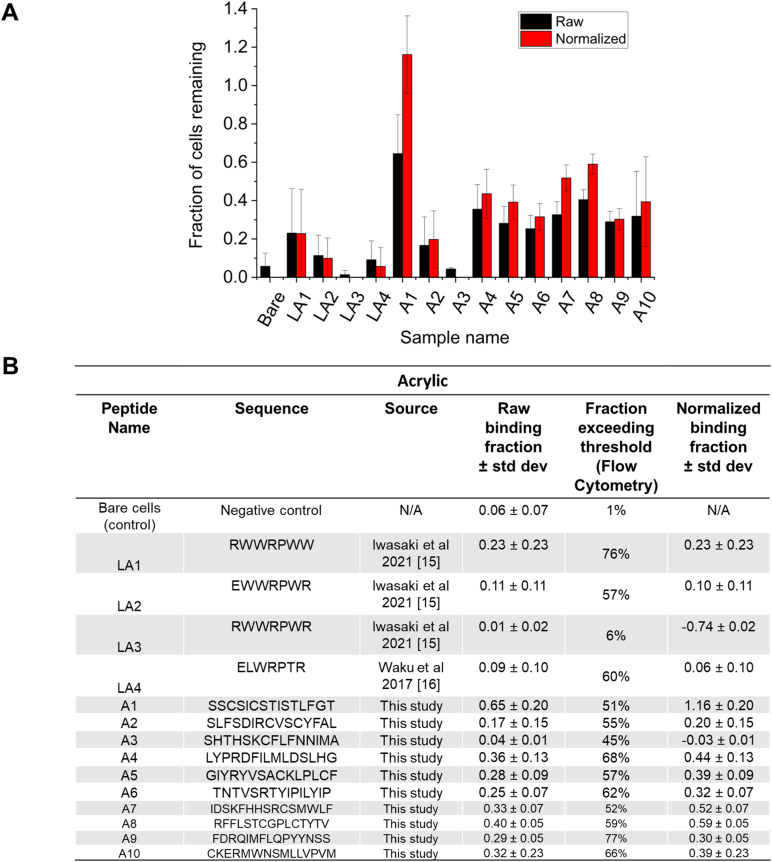
Acrylic binding evaluated by MAPS-D. The ten most-common peptides from the initial library screen against PMMA are titled A1–A10. These peptides, along with peptides obtained from literature (LA1–LA4) were evaluated using Ibidi microfluidic chips. (A) Shows raw, non-normalized data in black bars, and normalized data in red bars. (B) Shows the amino acid sequences of all peptides, as well as their source. The average of five technical replicates are shown, and error bars represent standard deviation. The flow rate was 70 μL min^−1^.

The results of the Microfluidic Assessment of Adhesion by Surface Display (MAPS-D) experiments are shown in Fig. 3 for PS and Fig. 4 for PMMA. In panel (A) of Fig. 3 and 4, the *X* axis shows each peptide by abbreviation, and the *Y* axis represents the fraction of cells that remained on the polymer surface after a shear force of 0.06 Pa was applied for five minutes. The solid blue bars represent raw data without normalization from the cytometer data, and the dashed orange bars represent data after normalization for surface display percentages as observed in the flow cytometer. In cases where the peptide display was good, but the percentage of cells remaining was lower compared to the negative control (*e.g.* LS1), the normalized fraction is negative, and this is an artifact of the normalization process. Such artifacts are omitted in panel (A) as the lower limit to the *Y*-axis is set to 0, but this artifact is shown in the table in panel (B) for completeness. Panel (B) of Fig. 3 and 4 gives the sequence for the peptides that were measured using this method. Notably, while normalization does change the fraction of cells thought to remain on the surface, it did not particularly change the rank ordering of the peptides; poor binders remained poor binders after normalization. Peptides identified through 4 rounds of biopanning compared favorably to those that were found in the literature for both PS and PMMA.

The relative abundance of a given peptide identified by next-generation sequencing did not correlate well with binding affinity of the peptide. For PS, the most common peptide was among the weakest binders. The use of more stringent wash conditions (higher amounts of surfactant, more vigorous stirring of the coupons) may reduce or eliminate this decoupling between prevalence and performance. These results provide an important cautionary lesson for other biopanning studies; next-generation sequencing data is frequently analyzed to see which amino acids are most prevalent in which positions, without doing additional functional testing of the individual peptides in the library. If the individual peptides that are found through biopanning are not tested, it is possible to draw the wrong conclusions about what amino acids are most important in which positions. MAPS-D provides a convenient way to functionally test the individual peptides identified through biopanning without needing to synthesize and purify them, though as cell libraries can sometimes propagate unwanted mutations, it is still best practice to retransform constructs of interest rather than relying on cells isolated from biopanning.^47^

### Evaluation at variable flow rates

**Fig. 5:**
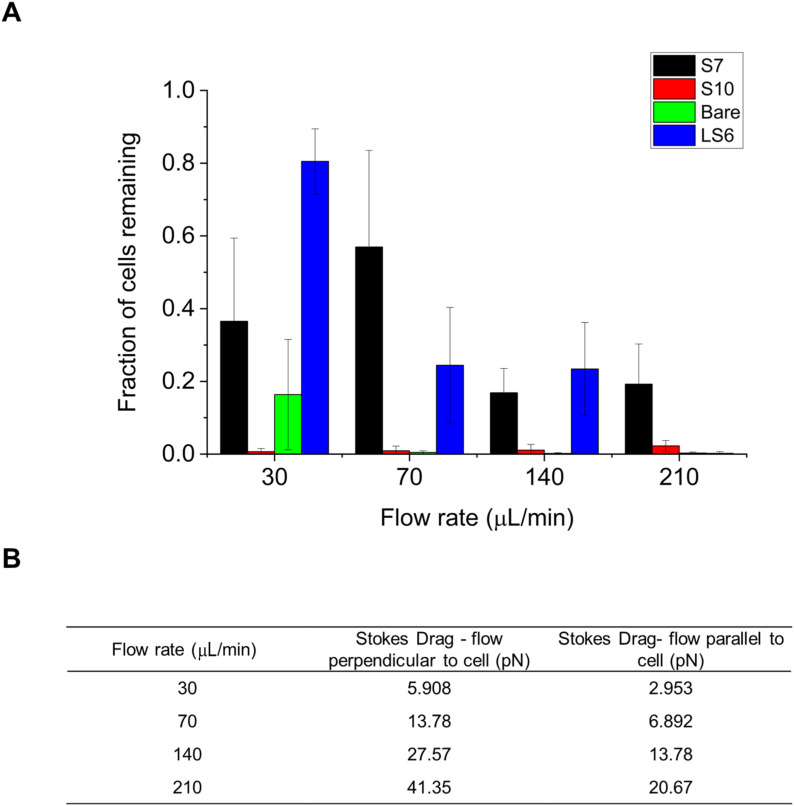
Peptide behavior at different flow rates. Peptides S7, LS6, S10, and a control cell expressing only GFP (“Bare”) were run on the Ibidi chips at flow rates of 30, 70, 140 and 210 μL min^−1^. (A) Shows the percentage of cells retained at each flow rate. (B) Shows the calculated Stokes drag force in piconewtons for each flow rate, assuming flow either perpendicular to the cell, or parallel to the cell. The average of four technical replicates are shown, and error bars represent standard deviation.

Three surface-displayed peptides (S7, LS6, S10) and a control of a cell that expressed just GFP were subjected to flow rates of 30, 70, 140 and 210 μL min^−1^, and the results are shown in Fig. 5. The drag forces and the shear forces faced by the cells were calculated using the flow cell geometry along with the employed volumetric flow rates assuming volumetric flow in the long side of cell (aligned normal to the flow) and in the face of a cell (aligned in the direction of flow). The calculated forces faced by the cells are shown in Fig. 5B.

As expected, the number of cells remaining is reduced at the higher flow rates (peptide S7 has a higher average number of cells remaining at 70 μL min^−1^ than at 30 μL min^−1^, but this difference is not statistically significant). Peptide S10 has very poor retention even at the lowest flow rate. Peptide S7 showed nearly 20% retention even at the highest flow rate. These results suggest that peptide LS6 has some releasing flow rate between 30 and 70 μL min^−1^, which corresponds to a Stokes drag force between 3.0 and 13.8 pN. It was found that bacteria expressing peptide S7 release at a flow between 70 and 140 μL min^−1^, corresponding to a drag force between 13.8 and 27.6 pN.

Clear differences in cellular adhesion were observed at different flow rates: two peptides, S7 and LS6 are difficult to differentiate at flow rates of 140 μL min^−1^ and below, but were very easy to differentiate at 210 μL min^−1^. However, to come to this conclusion, a large number of Ibidi chips had to be run. This argues for an improved microfluidic chip which would be able to measure multiple flow rates at once. A single chip with tapered channels, corresponding to different flow zones, would provide a large improvement in the amount of information that can be derived from MAPS-D experiments. Our group recently demonstrated the design and successful implementation of such a chip in a recent publication, which we believe could further increase the efficiency of MAPS-D.^49^

As the cells can be in a range of orientations relative to flow, the Stokes drag force calculations are presented in a range, representing the minimum and maximum cross-sectional areas that could be presented to the flow by individual cells. This is an approximate calculation, however it represents a good first estimate of the forces that each cell is capable of enduring, which is an important starting point for more-refined experiments and which may ultimately prove useful in the context of developing and testing an engineered biofilm. Deriving the exact force on each peptide using this method is difficult, as it is difficult to know exactly how many peptides are on the surface of any given cell. It is even difficult to quantify the exact number of peptides on a functionalized bead, which would otherwise be an attractive proxy for a cell. Further experimentation is required to improve the fidelity and quantitative value of these data, but provided the degree of functionalization of cells or beads can be accurately measured, it should be possible to find the forces acting on an individual peptide. To fully understand the true extent of these forces, a Multiphysics simulation (COMSOL or Ansys) approach could further enhance the veracity of these data.

### Validation of MAPS-D using synthesized peptides

**Fig. 6:**
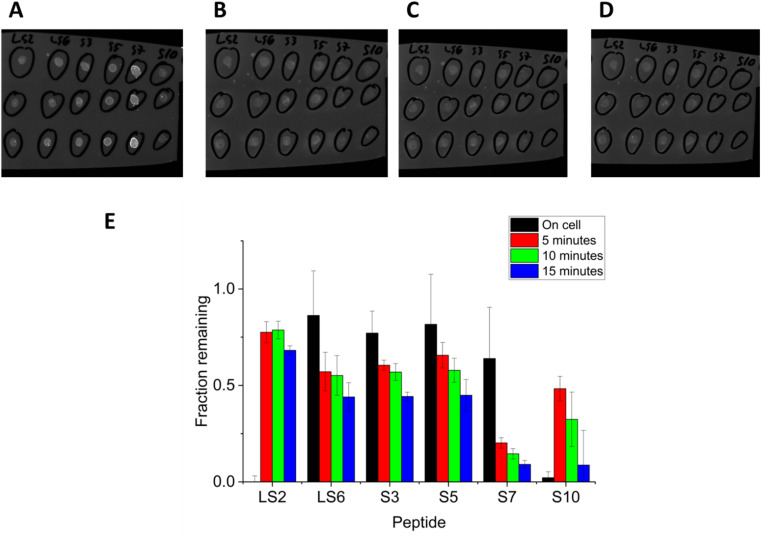
Evaluation of free peptide on polystyrene. Peptides from Fig. 4B were synthesized as free peptides, and functionalized with a C-terminal FAM dye. The peptides were spotted on a polystyrene coupon, then washed in a pan of DI water on an orbital shaker for varying amounts of time. (A) Shows the polystyrene coupon imaged before washing. (B–D) Show the same coupon after 5, 10, and 15 minutes of washing, respectively. The fraction of fluorescence for each peptide after washing is shown in the chart in (E). The first bar is the MAPS-D result of each peptide from Fig. 3, shown for purposes of comparison. The orange bar represents 5 minutes of washing, the gray bar, 10 minutes of washing, and the yellow bar, 15 minutes of washing respectively. Each bar represents the average of three spots, and the error bars represent standard deviation.

To assess the impact of the cell tethered to the peptide, a series of analogous cell-free peptides were prepared. The peptides were synthesized and dissolved in water, spotted onto a coupon of material, and then washed. A fluorescent tag on these “free” peptides helped determine how much of the peptide remained on the surface after washing. Six peptides that had been screened against PS were ordered from a commercial supplier (S3, S5, S7, S10, LS2 and LS6), representing a range of “good” and “bad” binders, from literature and from the novel binders presented here. The addition of a fluorescent dye to the C-terminus of the 15-mer peptides allowed for a fluorescent spot assay to be conducted on the PS surface: 50 μL of a 0.5 mg mL^−1^ aqueous solution of each peptide was spotted onto the surface, and the amount of fluorescence was compared before and after washing (Fig. 6). There was agreement between binding performance of cell surface displayed peptide and free peptide, provided that at least 60% of the cells were able to display the peptide on their surface. In Fig. 6, the peptides S3, S5, and LS6 have good retention both in cell surface displayed and free peptide conditions. Peptide S10 performs poorly under both conditions. The only discrepancies are the peptide LS2, whose performance in cell surface display was poor, but whose performance in the free peptide assay is excellent, and peptide S7, where performance on-cell is good but performance as a free peptide is poor. The discrepancy in LS2 is likely the result of the poor surface display of peptide LS2, with only 27% of the cells measured by flow cytometry appreciably displaying the peptide. The poor surface display may be the result of the high arginine content of this peptide: this charged residue is frequently found in antimicrobial peptides,^48^ and for that reason we speculate that peptide LS2 might be toxic to the cells. Correlation between on-cell and off-cell behavior was good for peptides that displayed on over 60% of cells measured by flow cytometry, suggesting the minimal threshold level for on-cell display required for MAPS-D to give meaningful data is between 27% and 60%. It may not be possible to realistically normalize for surface display if surface display is very poor. On the other hand, for the peptide S7, it is possible that the change from a buffered, salty system (medium) to a water medium may have changed the binding characteristics. Also, the fluorophore on the free peptide S7 was unusually bright compared to the other peptides, and this may have complicated the analysis.

### Advantages and disadvantages of MAPS-D

The key advantages of MAPS-D are that peptides can be rank-ordered for their affinity to potentially any substrate (here demonstrated with polymeric materials, one of which is opaque) in a straightforward method without the need to synthesize or purify the peptides. It is generally less expensive to purchase DNA than to purchase and purify peptides, and this advantage compounds when screening many peptides at once. While the MAPS-D technique only provides semi-quantitative data (*i.e.* it does not allow the direct calculation of a value such as *K*_d_), it does allow a useful rank ordering of peptides, enabling the identification and selection of the most promising candidates which can then be tested with more precise and quantitative methods. MAPS-D can also be applied to screen the impacts of point mutations on binding affinity, which may allow the correlation of specific positions/residues to binding activity. While this early demonstration of MAPS-D is still relatively low-throughput (about 25 replicates in an afternoon), the technique could be multiplexed on a microfluidic chip with many parallel channels. By marrying the foundational approach of a single flow-based shear assay with the miniaturization and automation of semiconductor-based microfluidic chip fabrication, a new realm of flow-based devices can be developed. These devices can feature a myriad of intricate, fluidically communicated channels that can massively parallelize tens, hundreds, or thousands of simultaneous experimental runs. Of course, the path forward should involve incremental verification and validation of these steps to scale up throughput, but the framework to do so is convincingly laid out in this work. The MAPS-D approach should be applicable to a wide range of substrates provided that the chip itself can be made sufficiently adhesive to contain the applied fluid and pressure.

The MAPS-D method also has some important limitations. First, running the method at a single shear rate may understate the true differences between the affinity of peptides for a given surface, as shown in Fig. 5: two peptides which may appear equivalent with an applied shear rate of 70 μL min^−1^ may be very different at a shear rate of 140 μL min^−1^. An improved microfluidic chip with channels designed for parallelized and multiplexed shear rate conditions was recently developed in our laboratory.^49^ A second limitation of the MAPS-D technique is that not all peptides will display equally well on a bacterial surface, requiring normalization by flow cytometry. It is also difficult to correlate flow cytometry data with an exact number of peptides present on the surface of an average cell. While techniques such as a Coomassie assay or an ELISA would give a sense of how many proteins are made by each cell, they would not be able to show how many proteins were successfully translated to the surface. It is therefore difficult to derive an exact force that causes each peptide to release from the surface. The effects of the number of peptides on a cell surface are also not clear; while intuitively having more peptides would seem to correlate with better binding, at higher surface concentrations of peptide there is the possibility of steric crowding or a reduction in the solvent accessibility of the peptide. In contrast, there may be avidity effects caused by having peptides in proximity to each other. However, normalizing for surface display did not produce an appreciable difference in the rank-ordering of the peptides in this experiment. When peptides display on at least 60% of cells, this technique allows a valid head-to-head comparison of several peptides at once. As flow cytometry can also be automated for high throughput, this process still represents a time and cost savings over synthesizing peptides and testing them individually using QCM, ITC, SPR, AFM, or SFA, each of which has its own idiosyncrasies and difficulties.

## Conclusion

A workflow for the MAPS-D approach to substrate screening was developed, demonstrated by biopanning experiments against polystyrene (PS) and polymethylmethacrylate (PMMA) and then evaluating the surface adhesion in a modest fluid flow field in a commercially-available microfluidic chip and normalizing the results with flow cytometry. The method identified a set of peptides whose performance compares favorably with other peptides reported in the literature, rank-ordered a series of peptides by binding affinity, and showed that the prevalence of peptides in biopanning does not always correlate to binding affinity.

The key advantages of the MAPS-D method are that it does not require expensive equipment, does not require synthesis or purification of peptides, can potentially be used across a wide range of substrates, and has the potential to be multiplexed and automated for increased throughput. The key limitations of the method are that not all peptides can be assessed using this method, as not all peptides will successfully display on a bacterial surface, and the information provided by the assay is somewhat imprecise as it is difficult to find the exact number of peptides displayed on a bacterial surface.

In future work, the MAPS-D technique could be used to identify additional promising peptide sequences that interact strongly with diverse substrates. More importantly, MAPS-D could be used to conduct directed evolution on peptides, and inform the understanding of the mechanisms by which peptides bind. By sequentially replacing each position of a peptide with an alanine residue (an alanine scan), the positions and residues which are most important to the adhesive function of the peptide could be revealed. This could in turn lead to the identification of hitherto-unknown peptide binding motifs which could be explored separately. The MAPS-D assay could also be used to validate computational predictions of potentially promising binding motifs, by making mutations that the computational model believes to be deleterious, and quickly assessing the effects of these mutations experimentally. Finally, MAPS-D provides a potentially attractive method for measuring the strength of cellular adhesion to a given surface, which may be relevant to the rapidly-emerging field of Engineered Living Materials (ELMs), which concerns itself in part with cellular interactions with polymer surfaces.

## Author contributions

MTK, JPJ, and JAO conceived and designed the study. RLR conducted next-generation sequencing and MMH did next-generation sequencing analysis. MTK and RAH designed and cloned plasmids. MTK and JAW carried out microfluidics experiments. JAW calculated the effects of different flow rates. MTK, RLR, MMH, JAW and JAO wrote the paper. MTK, JAW, and JAO edited the submission for publication.

## Conflicts of interest

There are no conflicts to declare.

## Supplementary Material

RA-015-D5RA02983J-s001

RA-015-D5RA02983J-s002

## Data Availability

The data supporting this article have been included as part of the SI. See DOI: https://doi.org/10.1039/d5ra02983j.
