# Supplementary material for: Microfluidic assessment of adhesion by surface display (MAPS-D): a novel method for evaluating peptide adhesion to polystyrene and poly(methyl methacrylate)
Source: RSC Adv. 2025 Sep 17;15(40):33854–67. doi: 10.1039/d5ra02983j (PMC12442031; doi:10.1039/d5ra02983j)
Supplement: RA-015-D5RA02983J-s001 [file RA-015-D5RA02983J-s001.pdf]

Supplementary Information (SI) for RSC Advances.
This journal is © The Royal Society of Chemistry 2025

SUPPORTING INFORMATION FOR:

Microfluidic Assessment of Adhesion by Surface Display (MAPS-D): A Novel Method for Evaluating Peptide Adhesion to Polystyrene and PMMA

Mark T. Kozlowski[1], Rebecca L. Renberg[1], Margaret M. Hurley[1], Jose A. Wippold[1], Justin P. Jahnke[1], Randall A. Hughes[1], Joshua A. Orlicki[1,*]

[1] DEVCOM Army Research Laboratory, Army Research Directorate, 2800 Powder Mill Road, Adelphi, MD 20783 USA

* Corresponding Author: joshua.a.orlicki.civ@army.mil

Materials and Methods

Materials

All gene blocks (Gblocks), primers, and deoxyribonucleic acid (DNA) duplexes were purchased from Integrated DNA Technologies (IDT; Coralville, Iowa). Restriction enzymes and anti-Histidine antibodies functionalized with AlexaFluor 488 were obtained from ThermoFisher Scientific (Waltham, MA) and T7 DNA ligase New England Biolabs (NEB; Ipswich, Massachusetts). Lysogeny broth (LB) medium was purchased in powdered form from SigmaAldrich (Milwaukee, WI), dissolved in deionized (DI) water according to manufacturer's instructions, and autoclaved for 15 min at 121° C. LB agar, for use in plates, consisted of LB medium, supplemented with 2% bacteriological agar (SigmaAldrich). Molecular-biology-grade water and glycerol were purchased from ThermoFisher.

2xYT medium was made by dissolving 16 g of tryptone, 10 g of yeast extract, and 5 g of sodium chloride (NaCl) in 1L of water. The medium was then sterilized by autoclaving for 15 min at 121° C. Glycerol-yeast-tryptone (GYT) medium was made by mixing 100 mL of glycerol, 1.25 g of yeast extract, and 2.50 g of tryptone per liter of molecular-biology-grade water, which was then sterilized by autoclaving. Super-optimal broth with catabolite repression (SOC) medium was made by first dissolving 20 g of tryptone, 5 g of yeast extract, and 0.584 g of sodium chloride (NaCl) per liter of distilled water, which was then autoclaved. Then 1M solutions of magnesium chloride ($MgCl_2$) and magnesium sulfate ($MgSO_4$) were made up and autoclaved separately. $MgCl_2$ and $MgSO_4$ were then added to the mixture of tryptone, yeast extract, and NaCl at a ratio of 100:1, such that the final concentrations of $MgCl_2$ and $MgSO_4$ in SOC were 10 mM.

Electrically competent *Escherichia coli* of strains DH10B and BL21 were made in-house by the following method. Cells were grown overnight at 37 °C in LB medium. This overnight culture was used to inoculate 100 mL of 2xYT medium in a 250-mL baffled Erlenmeyer flask. The cells were allowed to grow to an optical density (OD) at 600 nm (OD600) of 0.5. The cells were then placed on ice for 10 min, and then centrifuged for 6 min at 5000x g. The cell pellets were then resuspended in 100 mL of sterile 10% v/v molecular-biology-grade glycerol in molecular-biology-grade water and centrifuged again. This wash process was repeated with 50 and 25 mL of 10% glycerol. Finally, the cells were resuspended in 2 mL of sterile GYT

medium in molecular-biology-grade water. DNA was transformed into these cells using the transformation protocol detailed below.

Cloning

The cloning strategy shown here is adapted from DEVCOM-ARL Technical Report AR-TR-9312 (September 2021).

Polymerase Chain Reaction (PCR)

PCR was conducted with an AccuPrime Pfx DNA polymerase mix kit (ThermoFisher), according to manufacturer instructions and starting with 50 ng of DNA to be amplified. Thermal cycling was conducted on a Bio-Rad T100 instrument, with the cycles arranged thusly: 2 min at 95 °C, followed by 35 cycles of 15 s at 95 °C, then 30 s at 50 °C, and then 30 s per kilobase at 68 °C. After the 35 cycles are concluded, a final extension step is conducted for 5 min at 68 °C and then the sample is held at 4 °C. The resulting DNA is cleaned up using a Qiagen clean-and-concentrator kit following the manufacturer's instructions (Qiagen, Hilden, Germany). In brief, for each 10 µL of DNA-containing solution to be cleaned, 30 µL of DNA binding buffer was added. The column is then washed with 750 µL of wash buffer by spinning at 16000 xg for 30 s. The tube is then spun once, empty, at 16000 g's to remove any residual wash buffer, and the DNA is removed from the column by elution in 8 µL of molecular-biology-grade water. The concentration of DNA is then measured using a DeNovix DS-11+ spectrophotometer (DeNovix, Wilmington, Delaware) and kept on ice for transformation in electrically competent *E. coli* cells.

Electroporation

Transformation of all plasmids including the library into *E. coli* strains DH10B (for stock maintenance) and BL21 (for expression) cells was achieved by electroporation in a Bio-Rad Micropulser electroporator (Bio-Rad, Hercules, CA). Approximately 1 µg of assembled DNA was added to 200 µL of cell suspension kept on ice. The 200 µL of cells were then placed in an electroporation cuvette (Bio-Rad) with a 0.2 cm gap. The cells were then shocked with a 2.5 kV pulse and the time constant was noted. After the pulse, 1 mL of sterile, warmed SOC medium was immediately added to the cuvette and the cells were transferred to a sterile culture tube for recovery. The cells are allowed to incubate at 37° C for 1 hour unless otherwise noted. Then, 50 µL of cell culture was spread on an LB-agar plate supplemented with antibiotics as appropriate, and the plate is allowed to grow overnight at 37° C unless otherwise noted.

Removal of stop codons from library

An NNK library DNA duplex encoding 15 amino-acid-long peptides (15-mer) was purchased from IDT as MTK-G003, with the library flanked by BsaI restriction enzyme sites (i.e., BsaI site-$(NNK)_{15}$-BsaI site, where N = any DNA base and K = guanine or thymine). The NNK library codes for all 20 amino acids but has a reduced incidence of stop codons compared to a NNN library. To eliminate stop codons, the library is initially cloned into the plasmid pFES.2AB, which consists of an ampicillin-resistance cassette in a split intein system under a rhamnose-inducible promoter. If the inserted duplex contains a stop codon, this

should prevent full translation of the ampicillin-resistance cassette, meaning duplexes containing stop codons do not propagate in plasmids.

The duplex DNA could not be made long enough to contain both the BsaI restriction sites and the AarI restriction sites necessary for the two steps of inserting the library first into pFES.2AB and then inserting the library into a surface-display construct. For this reason, PCR was conducted using the duplex MTK-G003 and the primers pLibAmpF and pLibAmpR to add the BsaI sites, and cleaned up using a Qiagen clean-and-concentrator kit.

Next, 300 ng of this cleaned and concentrated PCR product was mixed with 150 ng of plasmid pFES.2AB in a PCR tube, along with 0.25 µL of BsaI, 0.25 µL of T7 DNA ligase, 1 µL of 10 mM adenosine triphosphate (ATP), 1 µL of enzyme Buffer G, and molecular-biology-grade water up to a volume of 10 µL (all from Invitrogen). This mixture is duplicated in eight different PCR tubes, which are assembled simultaneously in a Bio-Rad T100 PCR block (Bio-Rad Laboratories, Hercules, California) with the following heating cycles: 1 min at 37 °C and then 1 min at 16 °C, for 35 cycles. There was a final incubation at 37 °C for 1 h, followed by a denaturation step of 15 min at 85 °C, after which the temperature was lowered to 4 °C. After assembly, the library was cleaned up and concentrated using a Qiagen clean-and-concentrator kit following the manufacturer's instructions. Transformation was conducted using the electroporation protocol previously described with the following modifications: the SOC medium used for recovery was supplemented with 0.04% L-rhamnose (SigmaAldrich). The cells were allowed to incubate in the L-rhamnose supplemented SOC at 30 °C for 3 h, and then plated on a series of LB-agar plates supplemented with 50 mg/L of ampicillin and 0.04% L-rhamnose. An aliquot of the culture was also diluted 100- and 1000-fold, to be plated on separate agar plates, to determine approximately how many clones were obtained. Simultaneously, 10 pg of pUC19 plasmid (ThermoFisher) was transformed into a separate aliquot of cells to test for electrical competence of the cells. The plates were incubated for 2 days at 30 °C, and colony counts were conducted on the 100- and 1000-fold diluted plates. Based on colony counts, the estimated size of the library was between 10^5 and 10^6 members. Later sequencing indicated approximately 250,000 unique sequences. The transformations of pUC19 resulted in an observed transformation efficiency of approximately 10^9 colonies per microgram of DNA.

The plates were then scraped into 50 mL of LB medium supplemented with 50 mg/L of ampicillin. The cells were allowed to grow overnight at 30 °C and then miniprepped using a Qiagen miniprep kit according to manufacturer's instructions. The resulting plasmid prep was held at –20 °C until the library was to be removed from the plasmid and inserted into a surface-display construct. The resulting plasmid is known as pFES-Library. To transfer the library from pFES2-library, which enables it to be inserted into pAT-GFPdrop in the third and final assembly step, PCR was conducted using the primers LibOut-for and LibOut-rev. These primers are designed to add AarI restriction sites with appropriate sticky ends. The result of this PCR can serve as Block 5 in the assembly scheme shown in figure S1.

Golden Gate assembly

The Golden Gate strategy relies on the use of Type IIs restriction enzymes, which can have different sticky ends. Because assembly will only occur where sticky ends match, it is possible to assemble several fragments of DNA simultaneously, in the proper order. Golden Gate also has the advantage of combining the enzymatic digestion and ligation of DNA in a single step.

Golden Gate assembly was conducted by gathering, in a PCR tube, 100 ng of plasmid DNA (vector DNA), and 150 ng of any blocks to be inserted. To this was added 0.25 µL of restriction enzyme (AarI, BsaI, or BsmBI), 0.25 µL of T7 DNA ligase, 1 µL of 10 mM ATP, 1 µL of manufacturer's enzyme buffer (appropriate to the enzyme being used), and molecular-biology-grade water up to a volume of 10 µL. The tube was then thermally cycled for 1 min at 37 °C and 1 min at 16 °C, for 35 cycles. There was a final incubation at 37 °C for 30 min, followed by a denaturation step of 15 min at 85 °C, after which the temperature was lowered to 4 °C. Next, 1 µL of the assembly mix was transferred directly into 100 µL of electrocompetent cells, and electroporated in a 0.2 cm gap cuvette with a voltage of 2.5 kV. The cells are then immediately transferred into plain SOC medium, placed in a culture tube and allowed to recover for 1 h at 37 °C and 250 rpm. Then, 50 µL of cells are spotted and plated out onto LB-agar plates supplemented with 30 mg/L chloramphenicol unless otherwise noted. Plasmids were sequence-verified by GeneWiz/Azenta (South Plainfield, New Jersey) and successful clones were separated for further use.

Construction of surface-display construct

The autotransporter system for surface display of the library was cloned using a modification of the Modular Cloning (MoClo) toolkit developed by Lee and coworkers,[1] and further explained in the appendix of ARL-TR-9312.[2] The strategy is a Golden Gate assembly using compatible ends from the restriction enzymes AarI, BsmBI, and BsaI (all obtained from Thermo-Fisher). The cloning strategy is illustrated in Fig. S1, and the final construct is obtained after three separate assemblies. Figure S1 shows individual restriction sites with the four-base recognition sequence for each.

For Assembly I, Block 1 is obtained by PCR of the plasmid pYTK001 using the primers MTK-p1001F and MTK-p1001R. Block 2 is ordered as a custom DNA Gblock from IDT (MTK-G002). Blocks 1 and 2 are mixed with plasmid pYTK001, and a Golden Gate assembly is conducted using BsmBI. Because of the judicious selection of complementary ends, the result of Assembly I is a plasmid Intermediate 1, which exists on the pYTK001 backbone. The success of this cloning was confirmed by DNA sequencing (Azenta, South Plainfield, NJ). For Assembly II, Intermediate 1 is first removed from its plasmid by PCR using primers InsertSeq-for and YTKseq-rev. This PCR product is then mixed with Block 3 (obtained by PCR of plasmid pDSJR using primers araC-for and araC-rev)[3] and Block 4 (Type4Term), which was ordered as a Gblock from IDT. Intermediate 1, Block 3, and Block 4 are mixed with the plasmid pYTK-001_234_UTK_mRFP_DO, and assembled using a BsaI digest. The plasmid pYTK-001_234_UTK_mRFP_DO contains a constitutively-expressed red fluorescent protein (RFP) as a dropout cassette. A successful Assembly II resulted in green colonies on the plate, whereas an unsuccessful Assembly II resulted in red colonies. Several green colonies

were chosen, and successful assembly was confirmed by DNA sequencing. The result of a successful Assembly II is the plasmid pAT-GFPdrop. Finally, Assembly III was conducted using Block 5, inserted using AarI and pAT-GFPdrop as the plasmid. Block 5 either consists of the result of a PCR reaction of plasmid pFES-Library using primers LibOut-for and LibOut-rev or duplex DNA ordered from IDT that encoded for a specific peptide of interest.

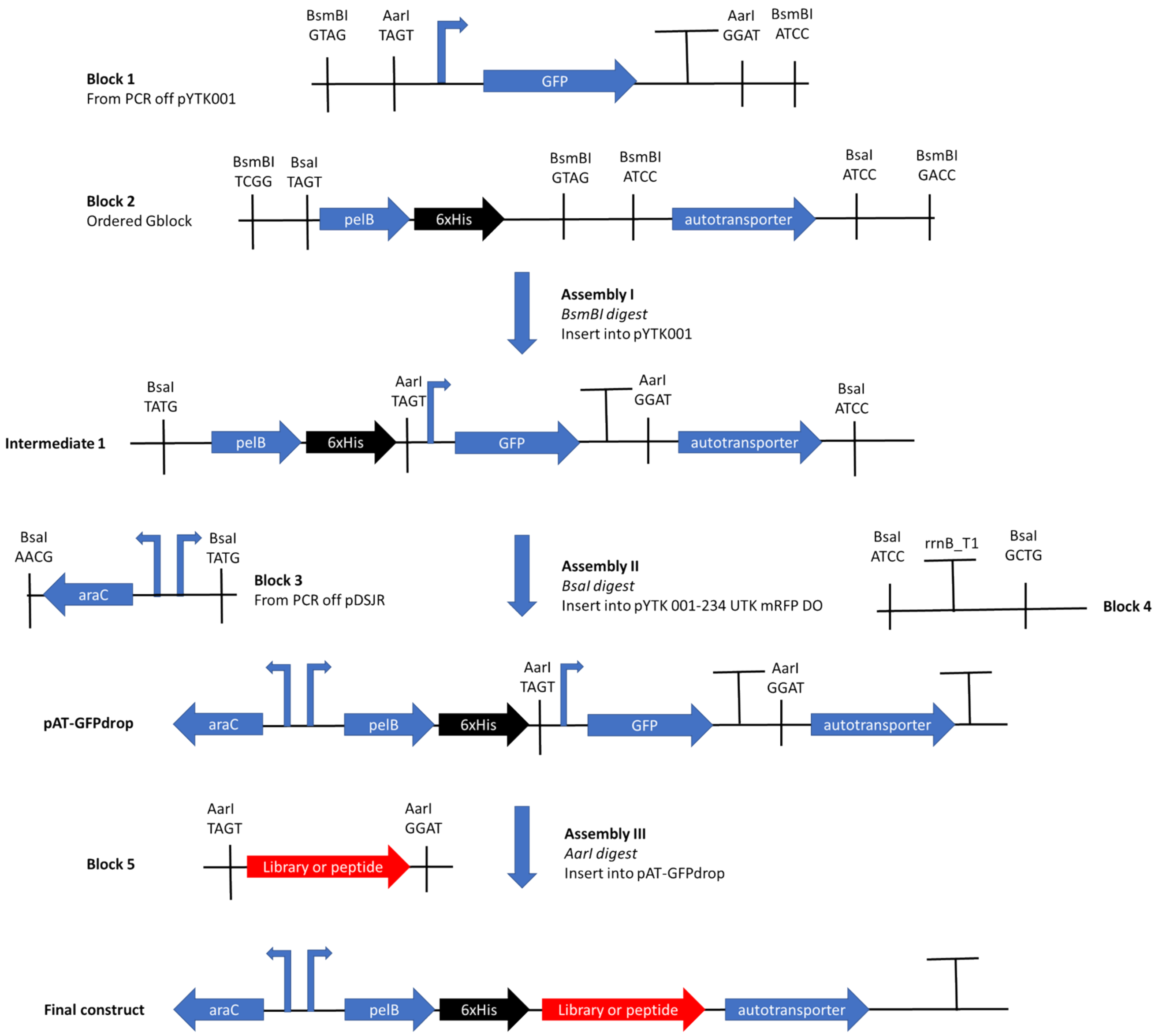

Figure S1: Cloning Strategy

Shown is the strategy for cloning the peptide library for surface display, as well as subsequent peptides for display. Downward-pointing arrows show Golden Gate assembly steps as Assembly I, II, and III respectively. The enzyme used in the digest is in italics. The vector (plasmid) receiving the insertion is below the enzyme. Each block that is inserted is named in bold, as well as its origin. Portions of the gene are shown as labelled arrows, and unlabeled arrows represent promoters. Restriction sites are labelled with vertical bars, as well as the recognition sites below the enzyme names.

Flow experiment protocol

Individual peptide display constructs were double-transformed with a plasmid that contained superfolder GFP (sfGFP) under a constitutive promoter (plasmid p15a-AmpR-TU2, generous gift of Nathan Schwalm, ARL) into *E. coli* BL21 cells by electroporation as previously described. The sfGFP plasmid had a p15a origin and ampicillin resistance cassette, to be compatible with the ColE1 origin and chloramphenicol resistance of the pYTK001-based backbones carrying the surface display constructs. The cells were allowed to recover in plain SOC medium for 1 hour at 37° C, then spread onto an LB-agar plate supplemented with 100 mg/L ampicillin and 30mg/L chloramphenicol for selection.

Individual colonies on the ampicillin/chloramphenicol plates were selected using a sterile pipette tip, and the colonies were dropped into 5 mL of fresh LB medium supplemented with 100 mg/L ampicillin and 30 mg/L chloramphenicol. These cultures were allowed to grow overnight at 37° C and 250 rpm. The following day, 200 µL of overnight culture was added to 5 mL of fresh LB medium supplemented with ampicillin and chloramphenicol. The culture was allowed to grow for 2 hours at 37° C and 250 rpm, then expression of the surface-display constructs was induced by adding sterile-filtered L-arabinose in water to a final concentration of 0.1% w/v (Sigma-Aldrich). The culture was allowed to grow for an additional hour at the same conditions, then approximately 500 µL of culture was added into the chips by micropipette. The cells were allowed to settle inside the chips for an additional hour at room temperature. Five technical replicates were conducted of each induced sample.

Initial fluorescence images were taken of the Ibidi chips using a Panthera C2 microscope with an FTIC imaging unit and an attached Moticam ProS5 lite camera at 10X magnification (Motic, Vancouver, BC, Canada). Imaging was done with top illumination and imaging as the PS sample was not transparent. One port of the Ibidi chip was then attached using the appropriate adaptor to a 10 mL syringe containing DI water that was placed in an Aladdin AL-8000 syringe pump (World Precision Instruments, Sarasota, FL). The syringe pump was set to dispense at a rate of 70 µL per minute. Water at room temperature was then flowed through the chip for 5 minutes. Without disconnecting the chip from the syringe, the chip was then imaged again in the same position as before.

Algorithm used for cell counting

Analysis of before and after images was conducted using ImageJ (National Institutes of Health). Images were first converted into 16-bit black-and-white images, then globally thresholded by eye to eliminate noise.

Batch processing of the thresholded images was conducted using the following script:

```
makeRectangle(15, 129, 1202, 612);

run("Crop");

setOption("BlackBackground", true);

run("Convert to Mask");

run("Make Binary");
```

```
run("Watershed");

run("Analyze Particles...", "size=3-15 pixel clear summarize");

close();
```

The cropping step was taken to eliminate a persistent reflection from the microscope turret that occurred in approximately the bottom third of the image.

Results from running the script were saved as .CSV and imported into Microsoft Excel. The number of particles from the images taken after flow was divided by the number of particles from the images taken before flow, to give a fraction of cells remaining after flow. These results were then gathered into histograms as shown in figures 3 and 4 in the main paper, where the bars represent the average of five technical replicates and the error bars represent standard deviation.

Tables: DNA ordered, and plasmids used

Table 1: Primers and Gblocks ordered

Name	Sequence	Purpose
Type4Term	gcatcgtctcatcggtctcaatcctaactcgagCACATCAGCCAGTGGAAGCCGAAGGTCCCGAACCGCGAGGACAAATACAAGAAGTAAagcggataacaatttcacacagATAAAACGAAAGGCTCAGTCGAAAGACTGGGCCTTTCGTTTTATgatccagtaatgacctcagaaGCTGTGAGACCTGAGACGGCAT	Block 4 of figure 1. rrnB_T1 terminator cloned as a Type 4 part
MTK-G002	gcatcgtctcatcggtctcatatgATGAAATACCTATTGCCTACGGCAGCCGCTGGATTGTTATTACTCGCGGCCCAGCCGGCCATGGCGCATCACCACCATCACCATACTAGTgGTAGtGAGACGaggctcagataCGTCTCtATCCGGTTCTGGCTCTGGTTCCGGTAGCGGTAGCGGTAGCGGCTCTGGTAGCGGTTCCATCGACAATTCAGCCGCAATTAGTATGGCAAATCCACGTCCACCAACACCGCGGacacctacaccgggtccggaccttaatgttgataacgatctgcgtccagaggcgggttcatatattgcgaatcttgcggcggcaaatacaatgttcacaactcgcttgcatgagcgcctgggtaatacttactataccgatatggtaactggcgagcagaaacagacgacgatgtggatgcgccatgaaggaggccataacaaatggcgcgatgggtccggtcaattaaaaacccagtctaatcgttacgtgcttcaattaggtggtgatgttgctcaatggtcccagaatgggtctgatcgttggcacgtcggcgtgatggcgggttatgggaacagcgatagtaaaactatttctagtcgtacagggtaccgtgcaaaagcttcagtcaatggatactccacaggtctgtacgctacttggtatgctgatgatgaatctcgcaacggagcatatctggattcatgggcccaatacagttggttcgacaatacggttaagggcgatgatttacaaagcgagtcttataaatcaaaaggattcactgcatcccttgaagccggttacaaacataaactggccgagttcaatggcagccaaggaactcgcaacgaatggtatgtgcagccgcaagctcaggtgacttggatgggggtgaaagcggataagcatcgtgaatcaaatggtacattggtacattctaacggcgacggtaatgtccaaacccgcttgggtgttaagacctggcttaagtcccatcataagatggatgacggtaaatcccgtgagtttcagccctttgtcgaggttaactggctgcataacagcaaagactttagtacttcgatggatggcgtttctgttacccaagatggggctcgtaatattgcggagattaaaacaggggtggaagggcaacttaatgctaacctgaacgtctggggtaatgttggtgttcaagtagcggaccgcggttataacgacacttctgctatggtcggaatcaagtggcagttctgacGATCCTGAGACCTGAGACGGCAT	Block 2 of figure 1. Autotransporter, with 6xHis tag, and space for insertion of GFP dropout.
MTK-G003	CTCCAGGTACACCTGCGTACTAGTNNKNNKNNKNNKNNKNNKNNKNNKNNKNNKNNKNNKNNKNNKNNKGGATGTTCGCAGGT	Library

	GGCTTCG	
LibraryOut-for	GGTGATGGATTGTCTGACAGG	Remove library from pFES.2AB
LibraryOut-rev	GTTTCACGAGTACCGATATTGTCC	Remove library from pFES.2AB
MTK-p1001F	CGCGTCTCTGTAGTTTTTGCAGGTGGGAAAGTGAAACGTGATTTC	Remove GFP from pYTK001 for use as a dropout, and add BsmBI restriction site
MTK-p1001R	GCGTCTCAGGATCCAAAAGCAGGTGTAAACGCAGAAAGGCCCACC	Remove GFP from pYTK001 for use as a dropout, and add BsmBI restriction site
pLibAmpF	GATACGGTCTCCAGGTACACCTGCGTACTAG	Initial amplification of library, adding BsaI sites
pLibAmpR	ATCTGGTCTCGAAGCCACCTGCGAACATC	Initial amplification of library, adding BsaI sites
araC-for	GCATCGTCTCATCGGTCTCAAACGtgctactccgtcaagccgtc	Remove araC and ParaBAD from pDSJR
araC-rev	ATGCCGTCTCAGGTCTCACATAacctcaaaggtaccgagctcg	Remove araC and ParaBAD from pDSJR
InsertSeq-for	GCTCTTCTCGCTAACCAAACC	Sequencing constructs in pYTK001 and dropout derivative
YTKseq-for	GCGTTATCCCCTGATTCTGTG	Sequencing constructs in pYTK001 and dropout derivative
YTKseq-rev	CGCTTGGACTCCTGTTGATAG	Sequencing constructs in pYTK001 and dropout derivative

Plasmids

Name	Antibiotic resistance	Purpose
pFES2.AB	Kanamycin<br>Ampicillin (split intein)	Remove stop codons
pDSJR	Chloramphenicol	Source for araBAD promoter
pYTK001	Chloramphenicol	Vector for Assembly I
pYTK001_234_UTK_mRFP_DO	Chloramphenicol	Vector for Assembly II
P15a-AmpR-TU2	Ampicillin	GFP marker for cells

Additional files

Plasmid maps of all five plasmids are provided separately, as GenBank format files (.gbk). The raw images used to derive the graphs are also provided.

References

(1) Lee, M. E.; DeLoache, W. C.; Cervantes, B.; Dueber, J. E. A Highly Characterized Yeast Toolkit for Modular, Multipart Assembly. *ACS Synthetic Biology* **2015**, *4* (9), 975-986. DOI: 10.1021/sb500366v.
(2) Kozlowski, M. T.; Hughes, R. A.; Pullen, R. M.; Orlicki, J. A. Peptide and Hydrophobin Interactions with Polymeric Substrates Screened by a Bacterial Surface Display Method. DEVCOM Army Research Laboratory: 2021; Vol. ARL-TR-9312.
(3) Dong, H.; Sarkes, D. A.; Rice, J. J.; Hurley, M. M.; Fu, A. J.; Stratis-Cullum, D. N. Living Bacteria–Nanoparticle Hybrids Mediated through Surface-Displayed Peptides. *Langmuir* **2018**, *34* (20), 5837-5848. DOI: 10.1021/acs.langmuir.8b00114.
